# Aqueous two-phase system (ATPS): an overview and advances in its applications

**DOI:** 10.1186/s12575-016-0048-8

**Published:** 2016-10-28

**Authors:** Mujahid Iqbal, Yanfei Tao, Shuyu Xie, Yufei Zhu, Dongmei Chen, Xu Wang, Lingli Huang, Dapeng Peng, Adeel Sattar, Muhammad Abu Bakr Shabbir, Hafiz Iftikhar Hussain, Saeed Ahmed, Zonghui Yuan

**Affiliations:** 1 National Reference Laboratory of Veterinary Drug Residues (HZAU)/MOA Key Laboratory of Food Safety Evaluation, Huazhong Agricultural University, Wuhan, Hubei 430070 China; 2 MAO Laboratory for Risk Assessment of Quality and Safety of Livestock and Poultry Products, Huazhong Agricultural University, Wuhan, Hubei 430070 China

**Keywords:** Aqueous two-phase system (ATPS), Biomolecule separation, Solvent extraction, Veterinary drug residues

## Abstract

Aqueous two-phase system (ATPS) is a liquid-liquid fractionation technique and has gained an interest because of great potential for the extraction, separation, purification and enrichment of proteins, membranes, viruses, enzymes, nucleic acids and other biomolecules both in industry and academia. Although, the partition behavior involved in the method is complex and difficult to predict. Current research shows that it has also been successfully used in the detection of veterinary drug residues in food, separation of precious metals, sewage treatment and a variety of other purposes. The ATPS is able to give high recovery yield and is easily to scale up. It is also very economic and environment friendly method. The aim of this review is to overview the basics of ATPS, optimization and its applications.

## History and background

In 1896, Martinus Willem Beijerinck accidently found the ATPS while mixing an aqueous solution of starch and gelatin. However, its real application was discovered by Per-Åke Albertsson. Since then, the ATPS has been used for a range of purposes [1–3]. These systems can be formed by mixing a variety of components in water [4]. But two-polymer and polymer-salt (e.g., phosphate, sulfate or citrate) systems have grown rapidly and a lot of work has been put into studying this technique using these types of ATPSs [2]. This method has advantages over conventional extraction techniques like, environment-friendly, low cost, capable of continuous operation, ease of scaling-up and is efficient for many kinds of experiments specially for the concentration and purification of biomolecules [1, 2, 4, 5]. The use of affinity ligands in ATPS can result in the higher recovery yields and higher purification folds of biological products as it is a primary stage recovery technique [6]. Affinity ligands can be covalently attached to polymer or polymer can also be modified with hydrophobic groups [5] Interested readers about aqueous two-phase affinity partitioning (ATPAP) are referred to an excellent review by Ruiz-Ruiz et al. [6].

Water as the main component of both phases in ATPS forms a gentle environment for biomolecules to separate and polymers stabilize their structure and biological activities [3, 4, 7–9] while other liquid-liquid extraction (LLE) methods could damage biological products because of the process conditions and organic solvents [1, 7]. The purpose of this review article is to overview the technique extensively and its applications in detail.

## Types of aqueous two-phase system (ATPS)

The most common biphasic systems are formed by two polymers (usually polyethylene glycol (PEG) and dextran) or a polymer and a salt (e.g., phosphate, sulfate or citrate). Other types include, ionic liquids and short-chain alcohols [1, 2, 4, 6, 7, 10, 11]. In addition to this, ionic and/or non-ionic surfactants are used for the formation of micellar and reverse micellar ATPSs [6, 12, 13]. Polymer – polymer/salt systems have been studied for more than five decades. Polymer – polymer systems are preferably used for the separation, recovery and purification solutes sensitive to the ionic environment as these systems pose low ionic strength. While, high ionic strength is the only disadvantage of polymer – salts system [14]. Alcohol – salt ATPS are inexpensive as compared to polymers and copolymers. These systems are also characterized by low viscosity, easy constituent recovery, and reduced settling times, but a major drawback of using this type of ATPS is that many proteins are not compatible with alcohol-rich phase [15, 16]. The aqueous micellar two-phase system was first introduced by Bordier for the separation of integral membrane proteins [17]. These systems are also useful for ionic environment sensitive solutes as nonionic surfactants can be used for the formation of these systems. Mixed micellar systems are becoming popular for showing selectivity features [18]. Most recently, ionic liquids (ILs) based ATPSs are being developed [19]. Poly-phase systems (three or four polymer phases) also have been constructed for the separation of biomolecules [7]. One-polymer ATPSs have also been reported, which utilize only one polymer for the formation of ATPS in water [20]. PEGs of different molecular weights are widely used polymers in ATPS due to their low toxicity, low price and low volatile nature [21]. Table 1 shows different types of ATPS with representative examples.

**Table 1:** Types of ATPS with representative examples

Types of ATPS	Representative examples			Reference
	Composition of ATPS	Product	Results	
Polymer – polymer	PEG – dextran	Chitinase	Successful partitioning of chitinase towards bottom phase	[161]
	PEG – dextran	Nanospheres, nanowires and DNA derivatized nanowires	Successful In situ binding Au nanospheres with Au nanowires	[162]
Polymer – salt	PEG – K_2_HPO_4_	B-phycoerythin	Recovery yield = 90 %	[163]
			Purification factor = 4	
	PEG 4000 – sulfate + 8.8 % NaCl	α-Amylase	Purification = 53 fold	[164]
			Purity = 86 %	
Alcohol – salt	2-propanol – K_2_HPO_4_	Lipase	Recovery yield = 99 % Purification factor = 13.5	[165]
	Ethanol – K_2_HPO_4_	2,3-butanediol	Recovery yield= >98 %	[16]
Micellar/reverse micellar ATPS	*n*-Decyl tetra (ethylene oxide)	Bacteriophages	Bacteriophages partitioning towards micelle poor phase	[12]
	Isooctane/ethylhexanol/methyltrioctyl ammoniumchloride	Plasmid DNA	Successful purification of DNA and RNA removal	[166]
Ionic liquids (ILs) – based ATPS	1-Butyl-3-methylimidazolium chloride – salt	Codeine and papaverine	Recovery yield= >90 % (codeine), >99 % (papaverine)	[167]
	Imidazolium – K_2_HPO_4_	Curcuminoids	Extraction yield = 96 %	[168]
			Purity= >51 %	

## Two-phase formation, thermodynamics and partitioning

Miscibility of solutions containing polymers is not a common phenomenon, this property of polymers results in the formation of two phases [3, 4, 7]. Similar incompatibility can be observed upon mixing a polymer and a high ionic strength salt. Polymer – polymer system forms large aggregates and because of steric exclusion, polymers start to separate between two different phases. In polymer – salt ATPS, salt absorbs large amounts of water and a same exclusion phenomena can be observed [5, 22].

Phase separation in ATPS is affected by different factors like, concentration and molecular weight (MW) of polymer, concentration and composition of salt [21, 23]. The presence of salt also influences phase behavior which also bring changes according to the type and concentration. Although, the mechanism through which salt influence ATPS in poorly understood [1, 7]. Generally, three forces: gravitational, flotation and frictional, act on a drop during phase separation and the balance between these forces determine its movement. The gravitational force depends on the density of drops while flotation and frictional forces depend on the flow properties of phases [10, 24]. Surface properties of materials and components of ATPS determine the partitioning between two phases [7]. Poorly understood partition behavior is a major barrier in widely adaptation of ATPS on commercial levels for the purification of biomolecules [25].

**Fig. 1:**
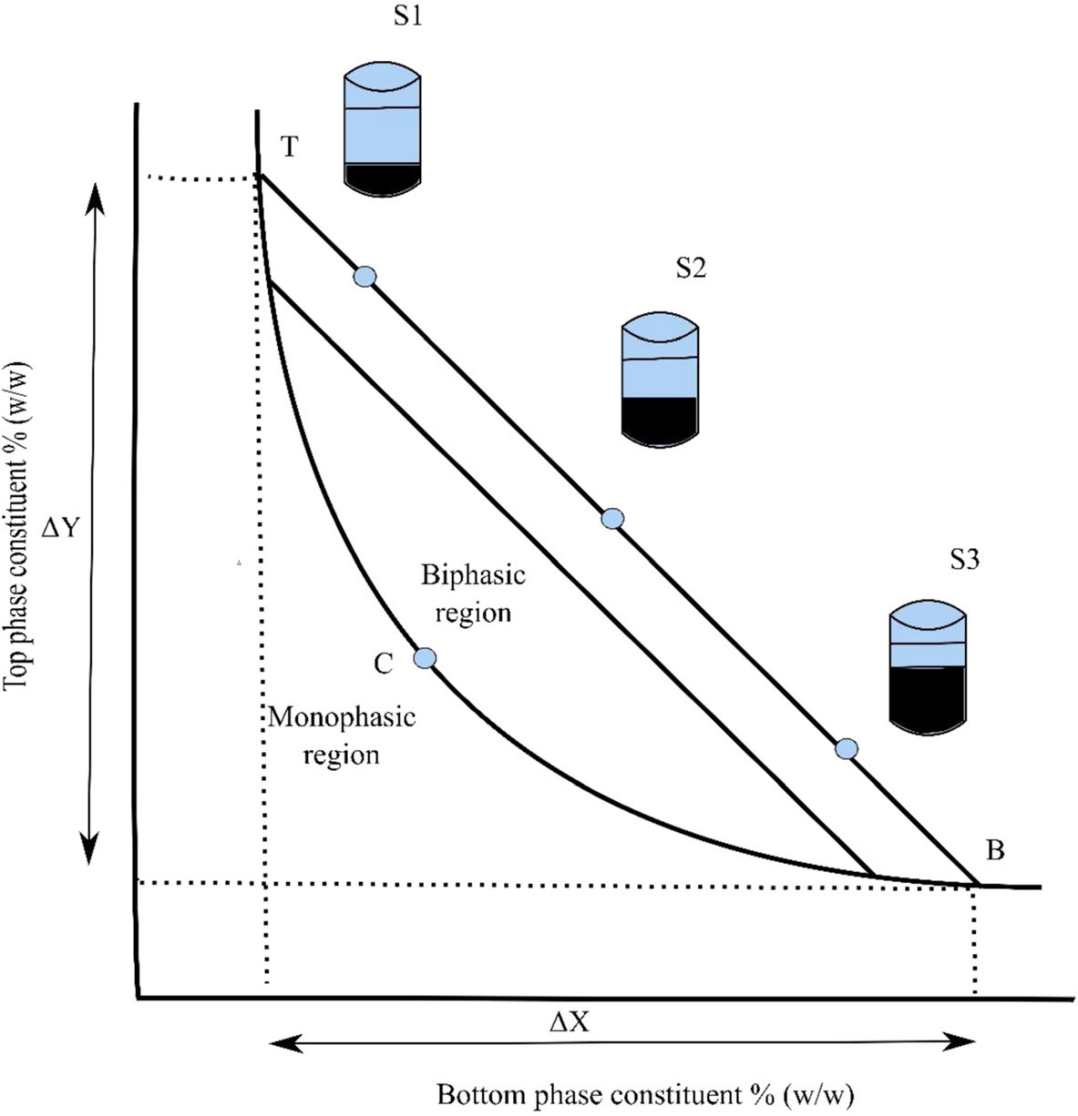
Schematic representation of phase diagram. Concentrations above binodal curve (TCB) forms aqueous two-phase system

Phase diagram (see Fig. 1) is like a fingerprint to a system under specific conditions (e.g., temperature and pH) which is unique and shows the potential working area of ATPS. It provides a set of information like concentration of components for two phase formation and their concentration in the top and bottom phases [4, 26]. The diagram (see Fig. 1) shows a binodal curve (TCB), which divides the region of component concentrations. This curve splits the concentrations which form two immiscible aqueous phases (above the binodal curve) from those that make one phase (below the binodal curve). The line (TB) in the diagram (see Fig. 1) is a tie line; it connects two nodes, which lie on the binodal curve. All the potential systems (e.g., S1, S2, S3) have same top phase and bottom phase equilibrium composition because of being on the same tie line. Point C on binodal is called as a critical point, just above this point the volume of both phases is theoretically equal. At point C the value tie line length (TLL) is equal to zero. The tie line length and component concentration has same units. The tie line length can be estimated by using the weight ratio as shown in equation below;

1 $$ \frac{V_t{\rho}_t}{V_b{\rho}_b}=\frac{SB}{ST} $$

Where V and ρ stands for volume and density of top (t) and bottom (b) phases while SB and ST are segments lengths as shown in Fig. 1.

Or by the analysis of top and bottom phase, which is a more precise method;

2 $$ TLL=\sqrt{\varDelta {X}^2+\varDelta {Y}^2} $$

The tie lines are commonly straight and the slope of tie line (STL) can be calculated with the help of equation 3. This is also helpful in the construction of further tie lines.

3 $$ STL=\frac{\varDelta Y}{\varDelta X} $$

Binodal can be determined by three methods; turbidometric titration method, cloud point method and node determination method [4, 26]. The researchers new to technique are referred to references [3, 4, 8, 27] for predetermined phase diagrams and methods for construction.

The equilibrium relationship between the top and the bottom phase of the ATPS determine the partition of biomolecules and the partition coefficient (*K*) is defined as;

4 $$ K=\frac{Conc{.}_{AT}}{Conc{.}_{AB}} $$

Where *Conc.*_*AT*_ is the concentration of component A in top phase and *Conc.*_*AB*_ is the concentration of A in the bottom phase at equilibrium [26]. So far, different models have been devised by the researchers to understand the partitioning in ATPS [25, 28–32]. There is no good comprehensive theory of liquid and liquid mixtures. In result, these models are based on the combination of different theories which makes a range of possible out-comes possible [33]. Albertsson’s model has been used commonly to describe ATPSs. He suggested six different kinds of partitions, each having a different kind of driving force [34]. According to his model, partition behavior is determined by these factors, separately or collectively and the manipulation of some of these factors would dominate the overall behavior.

i. Electrochemical – where electrical potential drives the partition
ii. Hydrophobicity – where hydrophilic properties of molecules and phases determine the separation
iii. Bio-specific affinity – this kind of partition occurs when required molecules binds to a specific site on polymer
iv. Size – molecular size or surface area of molecules is dominating factor
v. Conformation dependent – where partition depends on the conformation of the molecule

The logarithmic form of the factors of partition coefficient can be expressed in equation (5).

5 $$ \ln \kern0.5em K= \ln \kern0.5em {K}^o+ \ln \kern0.5em {K}_{elec}+ \ln \kern0.5em {K}_{hfob}+ \ln \kern0.5em {K}_{affinity}+ \ln \kern0.5em {K}_{size}+ \ln \kern0.5em {K}_{conf} $$

Where *elec* stands for electrochemical, *hfob*, *affinity*, *size* and *conf* denote as hydrophobic partitioning, affinity partitioning and conformation while *K* ^*o*^ include all other factors (e.g., environmental factors) [1, 3–5, 34]. Different theoretical and experimental models have been published by researchers. As, Andrews and Asenjo consider hydrophobicity as the main ruling factor of partition in polymer – polymer and polymer – salt ATPSs for protein [5, 35, 36].

### Factors influencing partitioning in ATPS

Since the partitioning mechanism in ATPS is still unknown. Most of the ATPSs are optimized according to the physicochemical properties of solutes of biomolecule. Different review articles [1, 5] and books [3, 27, 37–39] discuss these factors in detail. Main factors influencing partition behavior in ATPS are:

#### Molecular weight (MW) and concentration of polymer

As most of the ATPSs are composed of polymer – polymer/salt. MW of polymers greatly influence the partition. Generally,

- ↑ MW of polymer → ↓ concentration of polymer required for phase formation
- ↑ Differences between the MW of polymers → ↑ asymmetrical curve of the phase diagram
- ↑ MW of PEG → ↓ value of *K*

In a polymer – salt system, partition towards polymer-rich phase decreases upon increasing the concentration of polymer while in a polymer – polymer system partition decreases towards phase having high MW polymer. The main reason behind this phenomenon is the increase in the steric exclusion of biomolecule from that phase or because of changes in the hydrophobicity of phases [1, 5, 27] As increase in the MW of polymer increases hydrophobicity by reducing the hydrophilic groups/hydrophobic area [5].

#### Hydrophobicity

Hydrophobicity play an important role in the partitioning of protein. Two main effects: phase hydrophobicity effect and salting out effect, are involved in hydrophobic interactions [5, 40, 41]. In polymer – salt systems, hydrophobicity may be manipulated by varying TLL, MW of polymer and by adding a salt (e.g., NaCl). The low NaCl concentrations (<1 M) do not affect ATPS however, high salt concentrations (>1 M) changes the phase diagram [35]. The addition of salt in ATPSs has a significant effect on the partition coefficient [42]. These salts contain ions of different hydrophobicities and the hydrophobic ions force the partitioning of counter ions to phase with higher hydrophobicity and vice versa. The salting-out effect moves the biomolecule from salt-rich phase to polymer-rich phase [26].

#### pH

The pH of ATPS may alter the charge and surface properties of solute which affects the partitioning of biomolecule. The net charge of the protein turns negative in case of higher pH than the isoelectric point (pI) and positive if lesser than pI. If the pH is equal to pI, net charge will be zero [26]. It has been reported that the partitioning of negatively charged biomolecule in a higher pH system increases the partition coefficient and target biomolecule prefers top phase. Higher pH values than pI of protein induce an affinity towards PEG-rich phase because of the positive dipole moment [40, 43].

#### Temperature

Temperature greatly affects the composition of two phases in an ATPS, hence, the phase diagram. The changes in temperature also affect partition through viscosity and density. Therefore, it is always recommended by the researchers to have a strict control of temperatures in ATPS related experiments. In general, phase separation is obtained at lower temperature in a polymer – polymer ATPS with lower concentrations of polymer, however, an opposite effect is seen in polymer – salt system [27].

Partitioning behavior of biomolecule and phase separation rate is also influenced by the physico-chemical properties (i.e., density, viscosity and interfacial tension) of ATPS. Measurement of such properties have been explained by Albertsson [3], Zaslavsky [37] and Hatti-Kaul [4].

## Optimization of aqueous two-phase system

Since the partitioning behavior of biomolecules in ATPS is complex, many laborious trials have to be performed for the optimization of these systems. This optimization leads to an increased overall-cost [44]. One conventional way to optimize ATPS is a one-factor/variable-at-a-time (OFAT) in which specific factors are identified to study, but the major disadvantage of OFAT approach is not considering the interaction between the factors as the name indicate, one factor is studied at a time while keeping all factors constant. This usually results in the poor and false optimal conditions [26, 44, 45]. Nowadays, a multivariate statistical technique is used for the optimization of ATPS called “Design of Experiments (DoE)”. DoE consists of few experiments at a specific factor level combination [26, 44]. The general steps in a DoE are:

### Screening of variables

First step in a DoE process is the screening of significant factors (*k*), which demands further investigation because of their great influences on the out responses [26, 45, 46]. This is usually done by full factorial design (FFD) and fractional factorial design (fFD). In these designs all factors (*k*) are assigned two levels, high (+) and low (−). In FFD, experiments are carried out at different combinations of the factors with a total number of 2^*k*^. For instance, the number of factor is 2 (e.g., A and B). Then the possible number of experiments to be conducted is 2^2^ = 4 resulting in the combinations of (−,−), (+,−), (−,+) and (+,+). No doubt, this factorial design gives high accuracy results along with the possible interaction between the factors, but the number of experiments will be more in case of more factors (e.g., 2^5^) [26, 44–46]. To control this drastic increase in the number of experiments to one-half, one-quarter or a higher fraction of full fraction, fFD is used, which is denoted as 2^*k*-1^, 2^*k*-2^ and 2^*k*-4^. Another method used for the screening is Plackett-Burman design (PBD), a linear screening approach, used when only main influences are of interest [26, 44]. This could be represented in the form of the equation as follows

6 $$ y={\beta}_0+{\displaystyle {\sum}_{i=1}^k{\beta}_i{X}_i+\varepsilon } $$

(Here, y = predicted response variable, β_0_ and β_i_ = coefficient of regression, X_i_ = experiment factor and ε = random error).

Finally, in all three screening designs (FFD, fFD and PBD) the magnitude of the significant factors is analyzed by using analysis of variance (ANOVA) [26, 44, 47].

### Initial optimization

After the screening of factors/variables, the next step is to confirm the optimum level of these factors. As it is important to check that the factors are near to the optimal experimental region. This is done by an analysis of the model curvature after adding few center points experiments to screening model. Upon significant difference between these center point experiments and average out-put responses a model curvature occurs. This means the responses are situated in the optimal region and can be optimized in the next step which is not possible in case of no significant difference [26, 44, 45]. If no curvature exists, steepest ascent and steepest descent experiments are performed to increase or decrease the out-put responses to reach the proximity of optimal region [26, 44, 48]. Steepest ascent/descent experiments are useful in the determination of experimental direction. These experiments are initially performed at the center point of the significant factors and each factor level is increased or decreased in accordance with the magnitude of main effects. [26, 44]. In addition to this, these experiments have to be performed until no more increase in the out responses is observed, thus the general vicinity of the optimal experimental region can be drawn from the maximum response point of these experiments. Finally, these points can be taken as center points for final optimization [26, 44].

### Final optimization

Response surface methodology (RSM) is used for the final optimization of significant factors. Box and Wilson first reported this optimization approach [49]. This methodology is useful in the determination of optimal operating conditions and significant independent factors or their interactions with dependent output responses in the multivariate complex system (e.g., ATPS) [44, 50]. RSM is helpful in the prediction of responses by investigating an optimal experimental region and collecting experimental data which fits quadratic equation/second-order polynomial model [26, 44]. In this context, regression analysis is performed to select the best data representing equation and then output responses are analyzed by surface or contour plots [51]. Central composite design (CCD) and Box-Behken design (BBD) are two different multilevel designs of RSM but the CCD is most commonly used because of rotatability and uniform precision [45, 51]. CCD is also an extensively used model for the optimization of ATPS (PEG – Salt) [44, 51]. However, according to Raja et al., BBD has advantages over CCD, like, less number of experiments are done in BBD as compared to CCD and there are 3 factor levels in BBD while 5 in CCD [26]. Finally, the experimental data of these methods are used for fitting a full quadratic model and analyzed by regression analysis [26, 44].

### Analysis of model

After the optimization of significant factors in the previous step the quadratic equation obtained has to be solved analytically to determine the optimum values. Statistical software, for example Minitab®, Design-Expert® and Matlab®, which are being used in many fields for different applications [52–56], can also be used for this purpose. This analysis provides the optimum level of significant factors which maximize the response of model [26, 44, 45].

### Validation of model

In the final step of DoE experiments are performed using predicted values. Results are compared with those predicted values. If there is a small difference between predicted response and actual response, then the validation of the model is confirmed [26, 44, 46, 50, 57].

## Applications of aqueous two-phase system (ATPS)

### Proteins

Downstream processing is a very important step in the purification and separation of biomolecules in terms of cost. Specially, protein processing requires many steps for the purification because of complex starting material [1, 58]. Therefore, the demand for a high yielding and economical method for purification is increasing by the time. Chromatography of protein is not a suitable method to apply on a large scale due to batch processing and large pressure drops. Aqueous systems composed of organic solvents are not considered suitable for the purification of proteins because of their low solubility in these systems [5, 59]. To overcome such limitations, research is being focused on ATPS [58]. That’s why, the protein recovery from crude feedstocks at large-scale has been done by ATPS and this application of ATPS has attracted the most interest [7]. Protein partitioning in both phases mainly depends on the components of ATPS and their surface properties [5]. Mostly, protein will accumulate in top, hydrophobic and less polar phase, usually PEG. Proteins can be separated from one to another by changing MW of polymer, ion type or ionic strength in salt phase with the help of an additional salt (NaCl). These changes will affect the partition coefficient of protein partitioning.

Thaumatin is a low-calorie protein sweetener. In 1990, Cascone et al., studied the partition behavior of thaumatin from disrupted cells of *E. coli* in PEG – dextran and PEG – salt (phosphate) ATPSs. They investigated the effects of changes in phase forming components on partition coefficient *K* and observed that, MW of PEG, pH and the concentration of additional salt (NaCl) can alter *K*. While found no effect of protein concentration up to 40 gL^−1^. Analysis of protein was done by reverse-phase high performance liquid chromatography (HPLC). Purification conditions resulted in a 90–95 % recovery yield and 20-fold purification in a single step [60].

In order to find the partitioning behavior of biomolecule for the industrial scale-up ATPS, Diamond and Hsu published a generalized expression of protein partitioning in PEG – dextran ATPS by utilizing the modified version of Flory-Huggins theory. According to the expression, natural log of protein coefficient relates to the concentration difference of polymer between the two phases. The parameters of the relation were protein-water, protein-polymer and polymer-water interaction, function of protein and MW of phase forming polymer and the difference of electrostatic charge between phases. They also verified the relationship by observing the partition behavior of 17 different MW proteins [61]. In 1996, Hachem et al., concluded hydrophobicity as a dominant factor of partitioning pattern of proteins in ATPS, especially in polymer – salt (PEG – PO_4_) systems. They also observed that MW of proteins does not contribute in the partitioning, which confirms that attractive interactions (hydrophobic) between the polymer and proteins are responsible for protein partitioning [36]. Franco et al., established a method to study the effect of protein surface hydrophobicity on partitioning in ATPS. The experiments were conducted on two different series of hydrophobically modified proteins. They noticed the increase in the valve of *R* (separation power) upon the addition of NaCl in PEG – phosphate, which shows an important effect on system resolution for protein surface hydrophobicity. Protein surface hydrophobicity was twice as high when compared with the ATPS without NaCl. The same increase in the value of *R* was observed by Hachem and co-workers. Andrews and Asenjo, both were the part of these teams they strongly believe in the hydrophobicity as a single property on partitioning [35, 62]. Controlling partition of proteins by manipulating the temperature is a universally applicable method irrespective of the nature of the phases. Belval et al., discussed same phenomena in 1998, when they partitioned proteins in PEG – potassium phosphate system [63]. Liu and co-workers, utilized a nonionic surfactant *n*-decyl tetra (ethylene oxide), C_10_E_4,_ for the purification and concentration of proteins and viruses. This nonionic surfactant separates into two coexisting micellar phases in the water by an increase in the temperature. In their opinion, high water content in both phases and nondenaturing property of nonionic surfactant make this system potentially useful for purification and concentration of proteins and other biomolecules even at industrial scale [12]. Dodecyltriethylammonium bromide and sodium dodecylsulfate surfactants were used by Xiao et al., in 2000 for protein partitioning. They observed the concentration of negative charged BSA in surfactant-rich and positive charged lysozyme in surfactant-depleted phase in a cationic system while in anionic system opposite pattern of partitioning was observed [13]. Kresheck and Wang developed a new micellar ATPS for the separation of six typical globular proteins using *n*-dodecyldimethyohosphine oxide (APO12) and studied the effect of several factors [64]. Most of the previous studies explain partitioning on a molecular level or on basic thermodynamic theory, but, the kinetics of the separation process was ignored until Merchuk et al., reported their studies. They discussed the phase inversion in ATPS for protein separation which is very important for finding an optimal design in simulation schemes [65]. Fan and Glatz showed the charged protein (T4 lysozymes) partitioning in two polymer ATPS with salt effects as examination of charge effects is incomplete without considering the effect of salts [37]. They observed that increase in the concentration of salt shifts relatively more protein from the bottom to top phase and change in concentration of salt also influence electrostatic and non-electrostatic interactions [66].

“Smart polymers” are used for making one polymer ATPS instead of conventional two polymers or polymer/salt ATPS. Johansson et al., also used similar system for protein (lysozyme, bovine serum albumin and apolipoprotein A-1) purification. They used a linear random copolymer composed of ethylene oxide and propylene oxide (EOPO), hydrophobically modified at both ends with myristyl groups, (HM-EOPO). An ATPS composed of HM-EOPO forms a top phase of almost 100 % water and a bottom phase containing 5-9 % polymer. Their results showed the novelty of the system as it uses one polymer which can also be recovered and operated at moderate salt concentrations and temperature [20]. Alves and co-workers analyzed the partitioning of proteins from cheese whey, porcine insulin and bovine serum albumin (BSA). Results showed that α-lactalbumin (α-La) concentrate in PEG-rich phase and β-lactoglobulin (β-Lg) in saline phase in a system composed of 14 % PEG1500 – 18 % potassium phosphate. While in PEG1400 – maltodextrin 4000 (MD) system, β-Lg and BSA concentrate in MD rich-phase and porcine insulin showed affinity for PEG-rich phase [67]. Aqueous two-phase extraction (ATPE) has been used for the purification of proteins from different sources. Transgenic crops are utilized as expression hosts [68] and tobacco as a host produce recombinant protein, but the handling of its large biomass is a challenge. In order to develop a cost effective method to deal with huge biomass, Balasubramaniam et al., studied the purification of such proteins by ATPE with egg white lysozyme as a model protein. Their results declared ATPE a suitable method for partial purification of protein from tobacco [69]. Corn has also been used as an expression host for recombinant proteins. Gu and Glatz used ATPS for the extraction of recombinant protein from corn endosperm and germ [68]. Ferreira and co-workers showed the optimization of protein partitioning from *Zea mays* in PEG6000 – CaCl_2_ with the help of RSM. Results showed no influence of pH on phase diagrams and tie line length. RSM analysis showed, high pH and larger tie line length as favorable conditions for the recovery of proteins. The method was validated as the achieved partition coefficient (4.2) was in the range of theoretical partition coefficient (4.1–4.3). This process was also promising for continuous ATPSP due to the proteins (α and β-amylases) stability [70].

Protein extraction from urine can be useful for the diagnosis of several diseases (e.g., diabetes mellitus) and also in assessing the effectiveness of therapies. A healthy individual excretes less than 150 mg of protein in urine per day, but this may exceed to a few grams per day in renal diseases [71, 72]. In 2007, Wang et al., for the first time reported the extraction of protein from human urine with the help of IL-ATPS. They used an ATPS containing 1-butyl-3-methylimidazolium chloride (BmimCl) and K_2_HPO_4_. Protein was extracted into the BmimCl-rich top phase while contaminants were separated in K_2_HPO_4_ bottom phase [72]. Pei and co-workers also formed an IL (imidazolium) based ATPS to extract bovine serum albumin, cytochrome c, trypsin and γ-globulins. They observed the influence of various factors on the extraction efficiency and found that increasing temperatures and alkyl chain length IL increase the extraction. However, increase in pH slightly changes the extraction of cytochrome c. The system was found to be useful as the conformation of proteins was not affected because of the quick phase separation, lower viscosity and little emulsion formation. Thermodynamics studies showed hydrophobicity as the main driving force. Results depicted that 75–100 % extraction of proteins in IL-rich phase could be achieved in this system [73]. In 2008, Saravanan et al., observed the partitioning behavior of two model proteins (ovalbumin and myoglobin) in a two polymer (PEG – poly acrylic acid, PAA) ATPS and studied the influence of different factors. PEG4000 was found to be allowing better partitioning and the overall system was found to be suitable for low MW proteins. Moreover, 1 M NaCl, 20 ^o^C and a pH of 8.0 indicated better partitioning. The maximum yield of ovalbumin and myoglobin were 87.4 % and 95.2 % respectively. Overall the system was potential for the extraction of soluble proteins [74].

Several research articles regarding ATPS for protein partitioning have been published. Fisher et al., reported nanoparticle mediated protein separation in micellar systems with high recovery yields [75, 76]. IL-based ATPSs are being used in current scenario [77]. However, most recently, Li et al., showed the extraction of protein using deep eutectic solvent (DES) – ATPS. They selected Betaine-urea for extraction and studied the influence of pH, temperature, salt concentration, separation time, amount of protein and the mass of DES. More importantly, the conformation of protein was not changed and the extraction efficiency was 99.82 %. All the results suggested betaine-based DES – ATPS a potential new system for protein separation [78].

#### Enzymes

Conventional liquid-liquid extraction techniques are generally not applicable for the purification of enzymes due to the irreversible loss of enzymatic activity [27]. Other constraints of these traditional purification techniques are laborious procedures and low yields [79, 80]. Ultrafiltration and hydrophobic interaction chromatography are also multi-step, costly and low-yielding purification techniques. Affinity and ion exchange chromatography are the two most frequently used chromatography techniques for the purification of recombinant enzymes. But a sample pre-treatment step is required to facilitate the capture of the target protein. Ion exchange chromatography requires a desalting step while using LB media, which is the commonly used media and contain salt and yeast extract. Metal affinity chromatography is also impossible as yeast extract is known to contain cysteine rich protein [81]. Thus, ATPS was developed to overcome such limitations [82, 83]. It is also capable of combining different downstream processes into a single step [83, 84].

Microbial lipases are ubiquitous enzymes that are considered to be very important biocatalysts because of their substrate specifities and diversified properties [80, 85]. These enzymes are essential for a number of biotechnological applications. However, crude lipases contain isoforms and lipolytic proteins which results in the form of less biocatalytic reactions [80]. Ramakrishnan et al., studied typical polymer – salt (PEG – phosphate) ATPS for the extraction of an intracellular lipase from a lactic acid bacterium, *Enterococcus faecium*. In a PEG8000 – NaH_2_PO_4_ system result showed the partition towards sodium dihydrogen phosphate-rich bottom phase. After ultrafiltration, activity recovery was 5.99 % and purification factor of 82.09 % was achieved. In addition to this enzyme characterization study revealed that this enzyme was of alkaline nature and the MW of purified lipase was 19.2 kDa [80].

Laccases are another group of enzymes, which are found in plants, insects, bacteria and fungi. Fungal laccases have been purified and characterized extensively. They are successfully being used for pharmaceutical products (e.g., antibiotics, anticancer drugs, anesthetics and sedatives). Recently, in 2016, Rajagopalu et al., purified laccase from *Hericium erinaceusi* using PEG8000 – potassium phosphate ATPS and achieved a recovery 99 % [83].

Xylanases enzymes have been used in various applications, one of the major use is in the pulp and paper production as it can degrade the backbone of hemicellulose xylan [81, 86]. In 2016, Rahimpour et al., studied the two-stage purification of xyalanase using a 6 % PEG 6000–20 % phosphate system at first stage. Results showed about 78 % of recovery after final separation and a purification factors of 6.7 [81]. Packed columns are being used for enzyme purification because they require small space and are more efficient because of reduced axial mixing. A recovery of 94 % of xylanase was achieved by Igarashi et al., by PEG 4000 – dipotassium phosphate ATPS [87]. Elastase extraction from *Bacillus* s*p*. EL31410 was done by Xu and co-workers using an optimized ATPS of 23.1 % PEG2000 – 11.7 % KH_2_PO_4_/K_2_HPO_4_ with a predicted recovery of 89.5 % [57].

#### Monoclonal Antibodies (mAbs)

Antibody based therapies have played a key role in the treatment of infectious diseases, autoimmune disorders and cancers. Several antibodies have been approved by the US Food and Drug Administration (FDA) [47, 88]. This biopharmaceutical market has been steadily increasing since the day, FDA approved the first mAb (Orthoclone OKT3) and the global market has been projected to reach US$497.9 billion by the end of this decade [89]. Thus, these therapeutic agents have become very important and the demand of cost-effective, scalable and effective antibodies is increasing day by day. Improvements have been made in the upstream and downstream productivity as larger doses of mAbs achieve the required efficacy as compared to other therapeutic bio-products, such as vaccines, hormones and growth factors [90]. These purification improvements are also necessary to meet the purity standards of therapeutic molecules [88]. ATPS has proved to be a practical tool for the purification of mixture of biomolecules [47, 91]. Although, ATPE in the downstream processing of antibodies has been confined to the research studies only [90].

For first time, ATPS was used by Sulk et al., with thiophilic absorption chromatography for the isolation of IgG1 mAbs against horseradish peroxidase from hybridoma cell culture supernatant and recovery was 91 % after ATPE. The overall recovery was 71 % with a purification factor of 6.2 [92]. In 2007, Rosa et al., described the partitioning of immunoglobulins in two types (polymer—polymer and polymer – salt) of ATPS. Pure IgG was first extracted into a PEG-rich phase and then phosphate-rich phase. The purification factor was 2.7 and 5.9 respectively. They showed the purification of human IgG from an artificial mixture of proteins containing human serum albumin and myoglobin using PEG 3350 – phosphate with 100 % recovery and a purity of 99 % [88]. In another research work by the same authors, they reported the recovery of human IgG from Chinese hamster ovary (CHO) and hybridoma cell culture supernatant using PEG 6000 – phosphate [93]. An 88 and 90 % recovery yield in polymer rich phase were obtained, respectively. In 2009, Rosa et al. described that multistage equilibrium ATPE has advantages over single-stage experiment regarding recovery yield and purity. As 89 % of IgG was recovered in PEG-rich phase with 75 % of purity while in single-stage experiment 61 % recovery yield and 55 % protein purity were achieved. All factors (phase forming components, NaCl concentration, pH and volume ratio) were kept constant [90]. High concentrations of NaCl were found necessary for high recovery yields, but this may be a limitation during the scale-up. As, high concentration of NaCl is capable of damaging the equipment because of corrosive properties. The same year, Rosa and co-workers published another study of the downstream processing of antibodies using ATPS composed of PEG3350, dextran and triethylene glycol diglutaric acid (TEG-COOH). Single-stage and multistage strategies were evaluated and compared. In the single stage extraction system, they investigated the effect of pH, TEG-COOH concentration and volume ratio on the partitioning of antibodies in CHO supernatant and showed that high TEG-COOH concentrations and lower pH is suitable for the selective extraction of IgG in PEG-rich phase. Most productive conditions of single-stage extraction were 1.3 % (w/w) TEG-COOH and a volume ratio of 2.2 which resulted in 96 % recovery 87 % protein purity and a total purity of 43 %. The final IgG concentration was 0.21 mg/ml [94]. In order to increase both recovery yield and purity simultaneously, an affinity-enhanced multi-stage was simulated in which IgG was purified in a PEG-rich phase. This stage showed a final concentration of 1.04 mg/ml with a protein purity of 93 %. PEG/dextran ATPS was containing 1.3 % (w/w) TEG-COOH [94].

Upstream technology for the production of IgG has been considerably improved to gain higher titers and to keep this increasing, it is essential to solve the downstream processing that has been proving to be a bottleneck previously [93, 95, 96]. In this regard, Azevedo et al. studied the incorporation of ATPS, hydrophobic interaction chromatography (HIC) and size-exclusion chromatography (SEC) for the purification of IgG from a CHO cell supernatant. ATPS was composed of 10 % (w/w) PEG 3350 and 12 % (w/w) citrate, which allowed the concentration of IgG in citrate-rich phase with 97 % yield and 72 % protein purity and 41 % HPLC purity. This phase was then purified on phenyl-Sepharose HIC column using a citrate mobile phase. It resulted in the 86 % HPLC purity and 91 % protein purity. On third the step, SEC was allowed to final polishing. IgG aggregates were removed by injecting HIC-eluted fractions in a Superose 6 size-exclusion column. Finally, a 100 % pure IgG was obtained with 90 % yield [95].

Downstream processing can cost up to 80 % of the overall production cost [94] and phase separation by gravity in the downstream process is time consuming, which ultimately lead to an escalating production costs [6, 87, 97, 98]. To overcome this limitation column extractor are used which do not require centrifugation for phase separation. Rosa et al. evaluated the performance of a packed differential contractor with ATPE of IgG from a CHO cell supernatant. They selected PEG-rich phase as the disperse phase and the stainless steel as column packing bed after preliminary studies. The hydrodynamics of the stainless steel was also studied and the data were successfully adjusted to the Pratt-Thornton, Richard-Zaki and Mísek equations. The results showed the recovery yield of 85 % of IgG and 84 % protein purity [97]. In 2012, Silva et al. designed an ATPS in microfluidic device for the extraction of mAbs as, microscale process techniques are cost effective. They tagged IgG with fluorescein isothiocyanate (FITC) in an ATPS, composed of PEG/phosphate with NaCl. Fluorescence microscopy was used for the measurement of the phenomena of diffusion and partition of IgG from salt-rich phase to PEG-rich phase. These microscale results were found in accordance with macroscale results upon comparison except the reduction in the operation time [98].

A typical approach in the downstream processing of the mAbs includes concentration, selective purification and virus elimination. Selective purification is usually done by mAbs adsorption to a protein A resin and after that host cell proteins, DNA, IgG aggregates and virus removal are done by two chromatography steps [99]. But these chromatography steps have some low capacity, complex scale up and high cost of resins like limitations [99, 100]. To replace these chromatography steps by non-chromatography steps, Rosa at al. published a study which was easily scalable, capable of continuous operation and economic. A PEG – phosphate was developed for the extraction of IgG from CHO and PER.C6® cell supernatant. A process of extraction, back-extraction and washing were validated in a pump mixer-settler battery. Most of the impurities were removed during the extraction step. A global recovery yield of 80 % and more than 99 % of final protein purity from a CHO cell supernatant and from PER.C6® a 100 % global recovery yield was observed with a promising host cell protein/IgG ratio [99].

The reader is also referred to reference [96] for a comprehensive review of ATPE for the extraction and purification of mAbs. The authors have presented ATPE, a more economical and environmentally sustainable option as compared to currently established platforms specially protein A affinity chromatography. In this regard, with the aim of developing a non-chromatographic extraction method a hybrid process technology was investigated by Dhadge et al. in 2014. ATPS composed of PEG and dextran was added surface modified magnetic particles (MPs) at distinct salt concentrations. They observed that MPs coated dextran and gum Arabic expressed the lowest non-specific interactions and binding capacity of gum Arabic coated particles was excellent when modified with aminophenyl boronic acid. Recovery yield was 92 % with a protein purity of 98 %. MPs were also found to speed up the process of phase separation [101].

Silva et al. studied the purification of anti-CD34 in hybridoma cells by ATPS, using an integrated process that was capable to clarify and partially purify mAbs in a single step. Different ionic strengths (0–300 mM NaCl) was used to study, PEG – dextran systems at different pH values (pH 3,4 and 7). They also evaluated the effect of different MW of PEG (3350 and 600 Da). Results showed that ATPS composed of 7 % PEG 6000 Da, 5 % dextran 500 000 Da and 150 mM NaCl at pH 3 was the best in terms of recovery yield. This system was capable of to recover 84 ± 6.5 % IgG in the PEG-rich phase with 0.1 ± 0.2 % of cells [89]. Recently, in 2015, Muendges and co-workers figured out the solubility of biological material as the most limiting factor during ATPE. Keep in view, the decreased solubility of CHO cell supernatant proteins in the ATPS, high MW PEG was used in previous studies. Therefore, solubility of IgG in different phase forming components was screened and the best solubility was found in an ATPS composed of PEG2000/phosphate at pH six. The effect of NaCl was also investigated and it was observed that productive conditions were containing no or high amount of NaCl. Moreover, they concluded that the decrease in product-phase can improve the purity to a purification factor of up to 3.1 and recovery yield more than 90 % with a single step [102].

These studies suggest ATPE as an economical and environmentally stable option for the purification of monoclonal antibodies as compared to currently used protein A chromatography because of the high costs of resins, reusability limitations and formation of aggregates [96]. A selective comparison between ATPAP and protein A affinity chromatography for an almost similar recovery yield of IgG cited by Ruiz-Ruiz et al. would be worth mentioning here. Which shows the 91 % recovery and > 90 % purity of IgG in an ATPAP [103], and 95 % recovery with > 93 % purity [104] achieved by protein A affinity chromatography [6]. Although, a new generation of protein A resins has been developed with high binding capacities and high life span, but ATPE process has advantages especially when processing high titer cell culture supernatants [96].

### DNA and nucleic acids

The development of molecular biology and gene therapy is also dependent on the efficient and cost-effective isolation of DNA, RNA and other nucleic acid based biomolecules. Frerix and co-workers reported such low cost, polymer – salt (PEG – potassium phosphate) for the recovery of plasmid DNA (pDNA) in combination with ultrafiltration. ATPS was composed of 15 % PEG and 20 % potassium phosphate and strong partitioning of pDNA was observed at pH 7.0 [105].

A method optimized by RSM, showed the 99 % recovery of pDNA from an alkaline lysate of *Escherichia coli* with a system composed of PEG 400 – sodium citrate, 38.7 % of TLL, lysate load of 10 % and phase volume ration of 1.5 [50]. The experiments of Gomes at al., [106] resulted in almost 100 % recovery yield of pDNA with sodium citrate in a PEG – salt system and a lower recovery but higher purification in an ammonium sulfate based system. Finally, a mixture of 25 % (w/w) ammonium sulfate and 75 % (w/w) sodium citrate offered an optimum outcome with a 91.1 % recovery and 17.2 % purity.

Plasmid DNA displays a varied behavior in high molecular weight (HMW) PEG and in low molecular weight (LMW) PEG. In HMW-PEG based ATPS pDNA partition towards bottom phase only while in LMW-PEG ATPS partition of all phases with respect to the composition of the phase, temperature and lysate concentration used in ATPS [107].

Scaling-up of systems is very essential for the application of ATPS in industry to purify pDNA. Easy scale up of ATPS is one of the most advantageous side of this technique. A tenfold scale up was demonstrated recently with a recovery of 97.4 % of pDNA and an 86.4 % depletion of RNA [108]. Isolation of polymerase chain reaction (PCR) DNA fragments generated during in vitro PCR have also been reported [109].

### Virus, virus like particles

ATPS has been successfully employed for the recovery of virus, virus like particles (VLPs) or bionanoparticles however, has not been fully explored yet. Vaccine production, delivery vector for gene therapy and other applications of viruses and bionanuparticles demands high yielding downstream processing. ATPS is a very promising technique for the downstream processing of a range of biological products because of its ability to operate in a continuous mode. However, the integration of ATPS for the recovery of virus and virus to an industrial scale in still lagging because of complex partition mechanism [110].

Aqueous two-phase micellar system has been reported to be very efficient for the purification and concentration of viruses as viral particles have larger radii (100 Å to 2000 Å) than most of the proteins and according to excluded volume theory larger size protein exhibit strong partition behavior in micellar ATPSs [12].

Adenovirus was recovered from a crude lysate of HEK293 by Negrete et al., [111] using a PEG 300 – phosphate system, which yielded in 90 % of infectious particles [106]. ATPS was also investigated for the purification of VLPs (rotavirus-like particles), results showed a recovery of 85 % [112] and most recently single-stage and multi-stage ATPS has been employed for the purification of VLPs (human B19 parvo) from crude *Spodoptera frugiperda Sf*9 cell lysate. First time, a multi-stage ATPE was shown for VLPs, with satisfying results [113].

### Cells and organelles

Purification of whole cell and their organelles using aqueous two phase system have been investigated by several authors. There are many reasons behind the purification of these cells and their organelles. Like, platelets contain various growth factors (e.g., platelet-derived growth factor, transforming growth factor *β* and vascular endothelial growth factor). Thus, platelets are used as healing stimuli in different medical conditions. In 2006, Sumida and co-workers reported the separation of platelets from whole blood using 16 types of polymers in a polymer-based ATPS. They concluded that poly (2-methylacryloxyethyl phophorylcholine-co-*n*-butyl methacrylate) based separations were suitable for therapeutic purposes [114]. Separation of cells in ATPS depends on numerous intrinsic properties like, electrochemical charge, size, hydrophobic and hydrophilic surface properties [115] or in other words, interaction between the cell/organelle and polymer used in ATPS draw the separation pattern [116].

Nowadays microfluidic devices are used for the separation of blood cells, Toner and Irimia [117] excellently reviewed the use of such devices for blood cell separations. However, in 2009, for the first time a microfluidic separation method was applied to blood in an ATPS. Whole blood was exposed to PEG – dextran ATPS and the results showed a ratio of 9.13 of leukocytes to erythrocytes [118].

ATPS has also been used for several decades in plant related research. Numerous studied has been reported for the separation of whole-cell from cell lysates (e.g., isolation of plasma membrane vesicles from maize [119], purification of symbiosomes from pea [120] and selection of high yielding cells from cultured strawberry cells [121].

Extracellular vesicles such as exosomes and microvesicles are used as biomarkers for blood based diagnostic purposes. But there is a lack of effective purification strategies. Recently, in 2015, Shin et al., demonstrated an ATPS method for the purification of such extracellular vesicles. A polymer – polymer (PEG – dextran) ATPS was used which resulted in almost 70 % recovery just in a time span of 15 min [122].

### Low molecular weight compounds

Low molecular weight biomolecules (e.g., phytochemicals and secondary metabolites) are considered as high valued products due to their broad applications in food to pharmaceutical industry [123]. In traditional extraction and separation techniques, organic solvents are widely used, which are toxic and inflammable. This problem can be solved by the help of ATPS. However, there are only few studies regarding the application of ATPS for the processing these kind of biomolecules [123, 124].

In 2007, Chethana et al., [125] investigated the extraction and purification of betalains (derivatives of betalamic acids) from *Beta vulgaris*. As a natural food colorant, the demand of betalains is increasing, mainly because of its antimicrobial and antiviral activities. This differential partitioning resulted in the 70-75 % of betalains in top phase and sugars in bottom phase. Wu et al., showed the extraction of anthocyanins from mulberry (*Morus atropurpurea* Roxb.) Moreover, they stated that the ATPE did not alter the composition these natural pigments and the antioxidant activity of the extract was relatively high as compared to conventional extraction techniques [126]. Other reported studies include the recovery of crocins [127] from *Crocus sativas* using an ethanol-potassium phosphate ATPS and the extraction of anthocyanins [128] from *Brassica oleracea* L.

Recently, Zhang and his co-workers used ATPE for the extraction and enrichment of genistein and apigenin from pigeon pea roots (*Cajanus cajan* (L.) Millsp.). They employed an ATPS composed of 28 % ethanol and 22 % K_2_HPO_4_ and the recoveries were 93.8 % and 94 % for genistein and apigenin, respectively [124]. The extraction of natural products from plant matrix by ATPE is getting more importance due to increase in the demand in the nutraceutical industry.

### Drug residues in food and water

ATPS is also a novel technology for the separation and enrichment of drug residues in the water [129], food of animal origin [130] and herbs [131]. ATPS has several advantages over traditional organic solvent extraction, solid phase extraction (SPE) methods. It is regarded as an environmental friendly extraction procedure as both phases of ATPS contains water and no toxic volatile organic solvent is consumed in the process [129]. Moreover, the de-emulsification step is necessary in extraction methods (e.g., SPE, disperse solid-phase extraction (DSPE), quick, easy, cheap, effective, rugged and safe (QuEChERS) and others) for the extraction of analytes in milk because of the proteins and fats interference. But in ATPS it is possible to directly extract analytes in one single step [132].

Detection of residues at lower concentration is also possible with these biphasic systems. Han et al., showed the determination of chloramphenicol residues at concentrations lower than 1.5 μg kg^−1^, which was not possible in previously reported, dispersive liquid-liquid micro-extraction (DLLME) and matrix solid-phase dispersion (MSPD) methods [130]. Pesticides and herbicide residues may also accumulate in the animal derived foods (e.g., milk). Recently, Yang and his co-workers investigated five triazines herbicides in milk using ATPE. This simple ATPS was composed of acetonitrile and K_2_HPO_4_. The limits of detection (LOD) were 2.1, 2.6, 2.3, 2.8 and 2.5 μg L^−1^ for atraton, desmetryn, atrazine, terbumeton and terbuthylazine, respectively. The average recoveries of analytes were ranging from 86.3 to 120.6 % [132].

Roxithromycin (ROX) is a semi-synthetic antibiotic used frequently in human and veterinary medicine. Li et al. [129] reported the extraction of ROX residues in aqueous environment and proved that IL-base ATPS more efficient than traditional solvent extraction for ROX and other hydrophobic antibiotics. Table 2 is a compilation of studies reported so far, using ATPS for the extraction of drug residues in water and foods of animal origin.

**Table 2:** Extraction of drug residues in water and foods of animal origin using ATPS

Sample	Drug	ATPS	Detection limit	Average Extraction Efficiency (%)	Recovery (%)	Linear Range (μg mL^−1^)	Ref.
Water	Roxithromycin	1-butyl-3-methylimidazolium tetrafluoraborate – Na_2_CO_3_ (IL – salt)	0.03 μg mL^−1^	90.7	90.0–90.8	1.00–20.00 μg mL^−1^	[129]
Water	Sulfamethoxazole	Poly (propylene glycol)_400_ – NaH_2_PO_4_ (polymer – salt)	0.1 μg L^−1^	99.2	96.0–100.6	2.5–250.0 μg L^−1^	[169]
Lake Water	Chloramphenicol	1-butyl-3-methylimidazolium chloride – K_2_HPO_4_ (IL – salt)	0.1 ng mL^−1^	98.5	97.1–101.9	0.5–500 ng mL^−1^	[170]
Feed Water							
Milk							
Honey							
Feed Water	Chloramphenicol	1-butyl-3-methylimidazolium tetrafluoraborate – Na_3_C_6_H_5_O_7_ (IL – salt)	0.3 ng mL^−1^	90.1	90.4–102.7	2–1000 ng mL^−1^	[130]
Milk							
Honey							
Milk	Ciprofloxacin	Poly (ethylene glycol-ran-propylene glycol) EOPOL31 – K_2_HPO_4_ (polymer – salt)	6.8 ng g^−1^	1st = 97.7	1st = 83.5–90.2	-	[171]
Egg				2nd = 85.6	2nd = 83.8–86.8		
Shrimp							
Milk	Sulfonamides	1-butyl-3-methylimidazolium tetrafluoraborate – C_6_H_5_Na_3_O7.2H_2_O (IL – salt)	2.04–2.84 ng mL^−1^	-	72.32–108.96	8.55–1036.36 ng mL^−1^	[172]
Honey	Tetracycline (TC)	1-octyl-3-methylimidazolium bromide – sodium dodecyl sulfate (SDS)	TC = 5.8	-	85.5–110.9	TC = 20.1–301.2	[173]
	Oxytetracycline (OTC)		OTC = 8.2			OTC = 30.3–303.6	
	Chloramphenicol (CAP)		CAP = 4.2 μg kg^−1^			CAP = 20.4–305.4 μg kg^−1^	
Shrimp	Chloramphenicol	Polyoxyethylene lauryl ether (POELE10) – NaH_2_PO_4_ (polymer – salt)	0.8 μg kg^−1^	99.42	98–100.4	0.5–3.00 μg kg^−1^	[174]

### Metals and metal ions

ATPS is regarded as the most promising system also for the separation of metals and metal ions because of water being a major system constituent and the use of other non-inflammable and non-toxic constituents [4, 132, 134]. First paper regarding the partition of metal ions was published in 1984 by Zvarova and his co-workers [135]. In 1993, Roger et al. [136] published a review about separations of metal ions in ATPS and cited these early studies coming from Russian laboratories. Later, they investigated the effect of different factors (e.g., temperature and composition of ATPS) on the partition behavior of metal ions [34, 137–139]. Guzmán and Téllez reviewed the models involved in the affinity partitioning of metal ions, chapter is included in the book edited by Rogers and Eiteman [34].

Unlike the biomolecule partition, in which a wide range compounds was used for the formation of ATPS, most of the studies on metal partitioning show the ATPS formed by poly (ethylene oxide) (POE) and inorganic anions [134]. The discovery of triblock-copolymer opened new opportunities for metal extraction using ATPS. Triblock-copolymer such as (EO)_*x*_(PO)_*y*_(EO)_*x*_ aggregates in an aqueous solution at critical temperature and concentration. Micelles formed in the process of aggregation contain hydrophobic units like poly (propylene oxide) (PPO) at the core surrounded by hydrophilic units such as POE. These cores have the potential to solubilize the water insoluble agents and its hydrophobic metallic complexes [140]. Triblock-copolymer based ATPSs have been reported for the extraction and separation different metal and metal ions [133–135, 141, 142].

IL-based ATPSs are considered as the most “green” ATPS because of their properties of low volatility, non-flammability, strong solubility and large liquid range. Few studies have been reported regarding metal extraction employing IL-ATPS. Zheng et al., used an ATPS containing 1-hexyl-3-methyl imidazole dodecyl sulfonate and PEG6000 for the extraction of gold (III). They concluded ATPS as a promising technique for the extraction and separation of gold (III) [143]. Other studies include the separation of cadmium [144] and extraction of chromium [145] using ILATPS.

### Extractive fermentation

Extractive fermentation or in situ product recovery is the process in which ATPS is integrated with bioconversion to overcome the low product yield [146]. Low productivity is one of the most commonly observed issue in biotechnological processes because of inhibition, toxicity and instability of the end-product [4]. Therefore, about 60-90 % cost of a biological process is expended in downstream processing [147]. However, a high end-product concentration is feasible by extractive fermentation, because of low interfacial tension, continuous mode, selective separation and biocompatibility of ATPS [4, 147].

This strategy involves the continuous removal of product from its site of production/fermentation broth to the opposite phase simultaneously during production [26, 146]. Thus, the product of interest can be extracted from the system in a single step without performing biomass recovery or cell disruption [148]. A list of selected recent extractive fermentation studies using ATPS has been presented in Table 3.

**Table 3:** A list of selected recent extractive fermentation using ATPS

Product	ATPS	Organism	Ref.
Lipase	10 % EOPO	*Burkholderia cepacia*	[175]
Clavulanic acid	25 % (w/w) PEG8000 – phosphate salts	*Streptomyces* DAUFPE 3060	[176]
Lipase	9.6 % (w/w) PEG8000 – 1.0 % (w/w) Dextran T500	*Burkholderia pseudomallei*	[177]
Alkaline phosphatase	9.0 % (w/v) PEG4000 – 9.6 % (w/v) Dextran T500	*Bacillus lincheniformis* MTCC 1483	[178]
*β*-carotene, Lutein	6.6 % (w/w) PEG3350 – 8.4 % (w/w) Dextran 66900 And 4.22 % (w/w) PEG8000 – 9.77 % (w/w) Dextran 66900	*Synechocystis sp.* PCC 6803	[148]

### Environmental remediation

The applications of ATPS are not only limited to biotechnology, but also being extended to environmental remediation in various ways [4, 7].

Textile industry is responsible for the discharge of 10–15 % of annually produced 1 million tons of biodegradable resistant dyes. These dyes and their metabolites are toxic and carcinogenic in nature, thus posing a highly potential danger to aquatic biota and human health [149, 150]. Different physical and chemical methods have been studied for the removal of dyes from wastewaters. However, most of these methods are high cost, low efficient and laborious [151]. But these drawbacks could be overcome by ATPS which is an economic and eco-friendly method for the removal of textile dyes [152, 153]. Recently, Ferreira and co-workers [149] studied the IL-based ATPS for the extraction of a set of dyes in textile effluents and Ivetic et al. [150] investigated a PEG – salt ATPS model for Acid blue 9. Results showed ATPS as an excellent alternative dye removal method with high yield.

Hatti-Kaul [4, 7] cited Ström et al., [154] for large scale removal of microorganism from cutting fluids by using ATPS and demonstrated to be more effective than biocide treatment and irradiation. Other examples include the removal of metal ions [139], food coloring dyes [155, 156] aromatics from industrial and environmental settings [157].

### Other applications

ATPS has been most commonly used for the separation of macromolecules, however, it can also be employed as an analytical tool. The analytical applications (e.g., understanding chemical properties and behavior of protein, etc.) of ATPS are possible because the distribution between phases depends on system variation instead of the distribution of small molecules [4]. Or simply, partitioning in ATPS is sensitive to surface properties and conformation of phase forming component and particulate material [7]. According to Grilo et al. [1], ATPS is gaining interest as an analytical tool and 8 % of total application articles published in 2013 (ISI-indexed) account for analytical applications.

ATPS has been used with devices (column contactors) for continuous processes and microfluidics devices for the fractionation of biomolecules [9, 117, 118]. Moreover, ATPS can also be integrated with other separation techniques such as solvent sublation, which is termed as aqueous two-phase flotation [85, 158]. ATPS has also been successfully integrated with centrifugal partition chromatography (CPC) for the separation of biomolecules [150, 159].

Proteomics tools such as two dimensional electrophoresis (2DE) are being used with ATPS for the physicochemical characterization of biological samples, which is called as three dimensional (3D) proteomic analysis [160]. The combination of ATPS and dielectrophoresis offers a powerful separation and enrichment technique in which bio-particles are separated by ATPS and then concentrated by dielectrophoresis [160].

## Conclusion and future trends

ATPS is a simple, selective and low cost promising separation technique. Easy scalability of this technique makes it valid to be adopted by industries for downstream processing. However, it has been not widely used at commercial scale. The reasons behind this reluctance could be so many, but the poor understanding of the partition mechanism involved in the ATPS will always be on top. No doubt, the investigation and sorting out of the partition mechanisms governing ATPS will lead to a revolution in separation science. New types of ATPSs and more knowledge about phase forming components would result in more advance applications. Integration of ATPS with other promising tools will also be a breakthrough for recovering high value products.

## Abbreviations

2DE: Two dimensional electrophoresis
3D: Three dimensional
ANOVA: Analysis of variance
ATPAP: Aqueous two-phase affinity partitioning
ATPE: Aqueous two-phase extraction
ATPS: Aqueous two-phase system
BBD: Box-Behken design
BmimCl: Butyl-3-methylimidazolium chloride
BSA: Bovine serum albumin
CCD: Central composite design
CHO: Chinese hamster ovary
CPC: Centrifugal partition chromatography
DES: Deep eutectic solvent
DLLME: Dispersive liquid-liquid micro-extraction
DoE: Design of Experiments
DSPE: Disperse solid-phase extraction
EOPO: Ethylene oxide and propylene oxide
FDA: Food and Drug Administration
fFD: Fractional factorial design
FFD: Full factorial design
FITC: Fluorescein isothiocyanate
HIC: Hydrophobic interaction chromatography
HPLC: High performance liquid chromatography
ILs: Ionic liquids
LLE: Liquid-liquid extraction
LOD: Limits of detection
mAbs: Monoclonal antibodies
MPs: Magnetic particles
MSPD: Matrix solid-phase dispersion
MW: Molecular weight
OFAT: One-factor/variable-at-a-time
PBD: Plackett-Burman design
PCR: Polymerase chain reaction
pDNA: Plasmid DNA
PEG: Polyethylene glycol
pI: Isoelectric point
QuEChERS: Quick, easy, cheap, effective, rugged and safe
ROX: Roxithromycin
RSM: Response surface methodology
SEC: Size-exclusion chromatography
SPE: Solid phase extraction
STL: Slope of tie line
TEG-COOH: Triethylene glycol diglutaric acid
TLL: Tie line length
VLPs: Virus like particles
